# Diet with LPP for renal patients increases daily energy expenditure and improves motor function in Parkinsonian patients with motor fluctuations

**DOI:** 10.1080/10284150701414046

**Published:** 2007-09-25

**Authors:** Michela Barichella, Chiara Savardi, Andrea Mauri, Agnieszka Marczewska, Antonella Vairo, Cinzia Baldo, Arianna Massarotto, Sara Elisabetta Cordara, Gianni Pezzoli

**Affiliations:** 1 Parkinson Institute, Istituti Clinici di Perfezionamento, Milan, Italy; 2 Clinical Nutrition Unit, Istituti Clinici di Perfezionamento, Milan, Italy

**Keywords:** Parkinson's disease, diet, energy expenditure, levodopa

## Abstract

**Objective:**

To establish whether a diet based on the usage of low-protein products for renal patients (LPP) is associated with higher energy expenditure (EE) than a free low-protein diet (NO-LPP) by calculating 24 h EE by indirect calorimetry using an electronic armband monitor.

**Design:**

Randomized, cross-over, single-blind, pilot clinical trial performed comparing two different low-protein dietary regimens.

**Subjects:**

Forty-two days with LPP and 42 days with NO-LPP regimen in six patients with Parkinson's disease with levodopa.

**Methods:**

Monitoring patient response to two different nutritional schemes through indirect calorimetry (armband), BMI, Patient Global Improvement Scale.

**Results:**

Mean total EE was 1731 ± 265 kcal/day with NO-LPP vs. 1903 ± 265 kcal/day with LPP (*p* = 0.02).

**Conclusions:**

The usage of LPP increases EE and improves motor function in PD patients to a greater extent than NO-LPP dietary regimen. Calorie intake should be increased to prevent malnutrition in the long-term.

**Sponsorship:**

Fondazione Grigioni per il Morbo di Parkinson.

## Introduction

Parkinson's disease (PD) is a common movement disorder (worldwide prevalence: 3–4:1000), which develops in the second half of life and is characterized by bradykinesia, rigidity, resting tremor and postural instability (Zhang and Roman 1993; Quinn 1995). The disorder is the result of a neurodegenerative process that leads to the death of dopaminergic neurons in the substantia nigra located in the midbrain. The degenerative process is progressive and inevitably leads to major disability and morbidity associated with high healthcare expenditure (Schapira 1999). Its etiology has not been elucidated; it is believed that the neuronal degeneration is due to a number of environmental factors in genetically susceptible subjects (Sherer et al. 2001). Current therapy is symptomatic and consists in the replacement of dopamine, the neurotransmitter that the degenerated dopaminergic neurons no longer produce, by administering either a precursor of dopamine (levodopa) and/or other compounds that stimulate dopaminergic receptors (dopamine agonists); levodopa is the most effective replacement therapy and sooner or later it is added to the therapeutic regimen of all PD patients (Thanvi and Lo 2004). Also surgical symptomatic therapy exists, namely deep brain stimulation that consists in the stimulation of the damaged neuronal circuits via implanted electrodes; its use is confined to advanced cases that no longer respond to pharmacological therapy (Ahlskog 2001).

Most patients suffering from PD on treatment with levodopa experience frequent postprandial motor blocks, i.e. periods of loss of efficacy of pharmacological treatment, associated with a reduction in quality of life (Thanvi and Lo 2004).

The phenomenon has been ascribed also to the intake of amino acids during a protein-rich meal, which compete with levodopa, a neutral amino acid, for the same carriers during absorption from the gut and passage through the blood–brain barrier. Studies have shown that a low-protein meal at midday improves motor fluctuations and increases ON time (Juncos et al. 1987; Riley and Lang 1988; Carter et al. 1989; Duarte et al. 1993; Simon et al. 2004). Indeed, a low-protein diet is recommended by the guidelines for the management of PD (Olanow et al. 2001; Italian Neurological Society 2003).

In a previous 4-month study (Barichella et al. 2006) a diet with a controlled protein content (0.8 g/kg body weight) was compared with a low-protein diet based on the usage of low-protein food marketed for renal patients. The results showed that consumption of these foods reduced daily time in OFF and enabled a reduction in pharmacological therapy in some cases. A reduction in body weight during the first two months of consumption of the special food was observed. A possible explanation was that the improvement in motor function may have been associated with an increase in energy consumption that was not compensated by adequate calory intake. This hypothesis, however, was not clearly supported by evidence.

The objective of this study was to establish whether a low-protein diet based on the usage of low-protein food for renal patients (LPP) is associated with higher energy expenditure (EE) than a free low-protein diet (NO-LPP) by calculating 24 h EE by indirect calorimetry using an electronic armband monitor (Jakicic et al. 2004).

## Methods

This was a randomized, cross-over, single-blind clinical trial performed comparing two different low-protein dietary regimens. It was performed in the month of February 2006.

Six out of the 18 patients (30%) who took part in the previous study with low-protein food for renal patients were included (Barichella et al. 2006). The flow chart of the study is shown in Table I.

**Table I:** Study flow-chart.

Group 1 patients 1-3-5	LPP	Crossover	NO-LPP
Group 2 patients 2-4-6	NO-LPP		LPP
Evaluations	Day 0: baseline	Day 7	Day 14: end of study
Body weight	X		X
Height	X		
Body mass index	X		X
Nutritional status	X	X	
Mini Mental State examination	X		
Dietary instructions	X	X	
PGI		X	X
Diary dispensing	X	X	
Diary collection		X	X
Armband	X	X	X

They were PD patients diagnosed according to Brain Bank Criteria (Hughes et al. 1992) attending the ICP Parkinson Institute, on treatment with levodopa, who were experiencing post-prandial motor blocks of at least 30 min during the 5 h after the midday meal.

The patient population included three women and three men, median age 66 (50;76) years, mean body weight 64.3 ± 11.1 kg, body mass index (BMI) 24.1 ± 2.6 kg/m^2^, median duration of disease 21 (11;27) years, mean levodopa dosage 579 ± 293 mg/day; all patients were also receiving a dopamine agonist. No patients had dementia. Further details are provided in Table II.

**Table II:** Patient population details.

Patient	Sex	Age	Years of disease	kcal/day: 1st week	kcal/day: 2nd week	Weight (kg)	Height (cm)	BMI (kg/m^2^)	Levodopa dose (mg/day)
1	F	76	21	1523 with LPP	1550 NO LPP	59	151	25.9	450
2	M	69	11	1795 NO LPP	1800 with LPP	83	172	28.1	1000
3	M	58	27	1800 with LPP	1800 NO LPP	67	164	24.9	500
4	F	64	23	1660 NO LPP	1650 with LPP	54	159	21.4	250
5	M	50	11	2250 with LPP	2250 NO LPP	69	177	22.1	400
6	F	68	13	1800 NO LPP	1800 with LPP	54	156	22.2	875

All patients were examined by a physician specialized in nutrition and were interviewed by a dietician at baseline, after a Mini Mental State examination had been performed to exclude dementia. They were also interviewed by a dietician so that she could prepare a dietary regimen tailored to the tastes of the patient in terms of source of protein for the evening meal and sauce for the pasta at midday. Patients were weighed and their height was measured so that their BMI could be calculated. Calory requirements were calculated on the basis of basal metabolism estimated using the formula of Harris and Benedict (1919) and adding 20–30% according to reported physical activity.

Patients were randomized, using a randomization code prepared by the Nutrition service of the Parkinson Institute, to one of two low-protein dietary regimens:

— a low-protein dietary regimen (0.8–1 g/kg ideal body weight) achieved using low-protein food marketed for renal patients (LPP). These products (pasta, bread and milk to be used for breakfast and for lunch) were given to the patient by a physician specialized in nutrition. Their composition in
— a low-protein dietary regimen (0.8–1 g/kg ideal body weight) achieved by diminishing the consumption of protein-rich food and not resorting to the usage of any special kind of food (NO-LPP).

Both dietary regimens provided on average the intake of 31.2 kcal/kg ideal body weight (range 30.0–34.0 kcal/kg), with calories spread out throughout the day; they were both in compliance with the guidelines for healthy nutrition in the Italian population (Guidelines for healthy nutrition 2003).

Patients were given detailed instructions so that direct comparisons between low-protein food and common food could be made (see example of 1800 kcal diet in Table III showing the difference in terms of protein content). Each diet was followed for 7 days before assessments. The content of LPP and common foods used for patient dietary regimens in terms of amino acids competing with levodopa for absorption is provided in Tables IVA and IVB.

**Table III:** A comparison between dietary regimen of 1800 kcal with and without LPP products.

Diet 1800 kcal with LPP		Diet 1800 kcal NO LPP		
Proteins 10.1%		Proteins 13.9%		
Lipids 28.7%		Lipids 26.7%		
Carbohydrates 61.2%		Carbohydrates 59.4%		
	Breakfast			
Tea or coffee	S.Q.	Tea or coffee	S.Q.	
Jam	g 25	Jam	g 25	
Biscuits LPP	g 50	Biscuits	g 50	
	Lunch			
Pasta LPP	g 80	Pasta	g 80	
Vegetables	S.Q.	Vegetables	S.Q.	
Oil	g 15	Oil	g 15	
Fruits	g 150	Fruits	g 150	
	Snack			
Biscuits LPP	g 50	Biscuits	g 50	
	Dinner			
Bread	g 50	Bread	g 50	
Vegetables	S.Q.	Vegetables	S.Q.	
Oil	g 15	Oil	g 15	
Fruits	g 150	Fruits	g 150	
Monday	Meat g 150	Monday	Meat g 150	
Tuesday	Cheese g 100	Tuesday	Cheese g 100	
Wednesday	Fish g 250	Wednesday	Fish g 250	
Thursday	Two eggs	Thursday	Two eggs	
Friday	Fish g 250	Friday	Fish g 250	
Saturday	Legumes g 100	Saturday	Legumes g 100	
Sunday	HAM g 100	Sunday	HAM g 100	
	Diet 1800 kcal with LPP		Diet 1800 kcal with NO LPP	
	Proteins (g)	Percentage of protein (percentage total kcal)	Protein (g)	Percentage of protein (percentage of total kcal)
Breakfast	0.50	0.16	3.55	0.80
Lunch	4.75	1.00	16.23	3.60
Snack	0.50	0.16	2.51	0.61
Dinner	39.72	8.82	39.72	8.82
Total value	45.49	10.10	62.01	13.83

**Table IVA:** A comparison in protein content between LPP and common foods.

LPP products	Proteins (g/100 g)	kcal/100 g	Common foods	Proteins (g/100 g)	kcal/100 g
*Breakfast*					
Semi-sweet biscuits	< 1	449	Semi-sweet biscuits	6.6	418
Plain biscuits	< 1.35	488.5	Plain biscuits	7.4	493
Wafers	< 1.2	539.5	Wafers	7.1	454
*Lunch*					
Breadsticks (grissini)	< 1.4	419	Breadsticks (grissini)	12.3	433
Toasted bread	< 1.6	399	Rice	7	362
Melba toast	< 1	421	Melba toast	11.3	410
Pasta	< 0.7	354	Pasta	10.8	356

**Table IVB:** Comparison between LPP and common foods in terms of content in aminoacids competing with levodopa for absorption.

LPP products	Phe (mg/100 g)	Tyr (mg/100 g)	Trp (mg/100 g)	Leu (mg/100 g)	Iso (mg/100 g)	Val (mg/100 g)	Common foods*	Phe (mg/100 g)	Tyr (mg/100 g)	Trp (mg/100 g)	Leu (mg/100 g)	Iso (mg/100 g)	Val (mg/100 g)
*Breakfast*							*Breakfast*						
Semi-sweet biscuits	< 60	< 60	–	–	–	–	Semi-sweet biscuits	644	462	133	1141	625	710
Plain biscuits	< 80	< 55	–	–	–	–	Plain biscuits	–	–	–	–	–	–
Wafers	< 45	< 35	–	–	–	–	Wafers	–	–	–	–	–	–
*Lunch*							*Lunch*						
Breadsticks (grissini)	< 40	< 20	7.7	36.1	18.7	26.8	Breadsticks (grissini)	–	–	–	–	–	–
Toasted bread	< 50	< 50	8.4	39	20.2	28.9	Rice	360	228	84	590	306	438
Melba toast	< 60	< 40	8.6	41.7	21.9	30.7	Melba toast	595	297				
Pasta	< 30	< 30	3.7	60.3	19.6	26.3	Pasta	542	310	105	834	455	544

* From: “Tables of food composition” Istituto Nazionale di Ricerca per gli Alimenti e la Nutrizione.

All assessments were made by staff blind to the dietary regimen of the patient, who was instructed not to mention it to the examiners.

Patients were given study diaries and were instructed to write down the following information everyday: hours of sleep; waking hours in ON, i.e. times when medication was working and motor symptoms are controlled (with and without dyskinesias) and hours in OFF, i.e. times when the medication was not working and symptoms reappeared; time of antiparkinsonian drug intake; time of meals; any deviations from the dietary regimen.

An armband (Bodymedia Sensewear Pro2) was positioned on the right triceps of the patients for the whole 14 day period (24 h per day) of the study, so that it could measure EE continuously. The SenseWear Pro Armband™ (Body Media, Pittsburgh, PA) is a newly developed commercially available device to assess EE. It has already been extensively used for research purposes and its use has been validated not only for usage in sports medicine (Fruin and Rankin 2004) and in particular environments, such as under water, but also during normal daily activity (Mignault et al 2005; Levine and Foster 2005). The device is worn on the right upper arm over the triceps muscle and monitors various physiological and movement parameters. Data from a variety of parameters including heat flux, accelerometry, galvanic skin response, skin temperature, near-body temperature and demographic characteristics (gender, age, height and body weight) are used to estimate EE utilizing proprietary equations developed by the manufacturer. Due to its lightness and wear ability, the armband monitor is particularly suitable for continuous patient monitoring for several days. The software data analysis was carried out at the end of each of the 7 days of the dietary regimen period.

At the end of each dietary regimen the patient global improvement (PGI) questionnaire was given to the patients, who completed it by themselves. The PGI served as an independent, yet patient-based assessment of a treatment effect.

The primary endpoint was EE. The secondary endpoints were: 24 hOFF time, 24 h ON time with and without dyskinesias.

The statistical analysis compared data related to days on balanced diet with data related to days on LPP diet using ANOVA.

Diary cards were coded with the number of the patient and the allocated sequence (AB or BA). The person who analyzed the data was blind to sequence.

## Results

All six patients completed the study as per protocol and provided 84 valid diaries, 42 with LPP and 42 with NO-LPP regimen.

### Diary results

**Figure 1:**
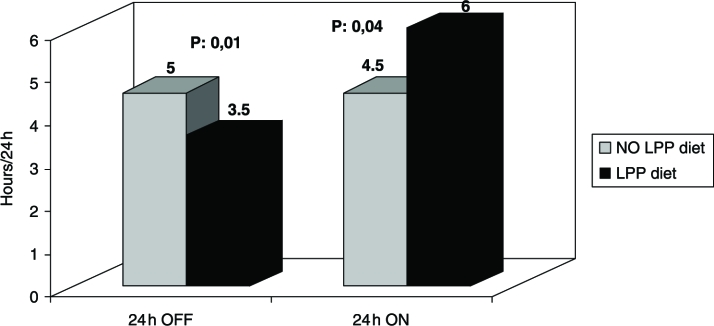
Diary results: 24 h OFF time is significantly shorter after LPP diet than after NO-LPP diet (*P* = 0.01); 24 h dyskinetic ON time is significantly longer after LPP diet (*P* = 0.04).

Twenty-four hours OFF time was significantly shorter after LPP diet than after NO-LPP diet (3.5 h vs. 5 h, *p* = 0.01); 24 h dyskinetic ON time was significantly longer after LPP diet (6 h vs. 4.5 h, *p* = 0.04) (Figure 1).

### Armband results

The armband was worn by the patients for 98% of the time in both the evaluation periods associated with the two nutritional schemes.

The daily hours of sleep were similar in the two groups (7.68 ± 1.94 with NO-LPP vs. 8.02 ± 2.2 h with LPP). These results are consistent with the sleep hours estimated from the patient diary analysis.

An increase in total EE of about 10% was noticed for the LPP dietary regimen compared to the NO-LPP diet: mean total EE was 1731 ± 265 kcal/day with NO-LPP diet vs. 1903 ± 265 kcal/day with LPP (*p* = 0.02). Also the time spent in physical activity was longer with LPP than with NO-LPP diet (1.75 ± 1.33 vs. 1.38 ± 1.32 h; *p* = 0.05).

### PGI results

According to PGI questionnaires, all patients expressed a benefit with LPP regimen (very much better *n* = 2, much better *n* = 4) while no benefit or worsening were expressed with the NO-LPP diet.

## Discussion

This study shows that the LPP dietary regimen is associated with a significant increase in EE in fluctuating PD patients, as measured by the armband. This finding is consistent with the additional evidence of improvement in motor function in such patients, expressed as a significant reduction in 24 h OFF time, according to both armband and patient diary data.

The only difference between the two low-protein dietary regimens (one using low-protein food for renal patients and the other being a free low-protein diet) was the midday meal protein content (with lower protein content for the low-protein product food nutritional scheme); the protein intake in the evening meal was the same. It appears that the increase in EE and greater improvement in motor function with LPP was due to better absorption of levodopa at midday, less hindered by lower protein intake. Calorie intake and hours of sleep were similar in the two groups and should not have influenced results. Furthermore, these results suggest that the evening meal does not play an important role in determining motor performance.

An additional finding was the increase in ON periods with dyskinesias, according to the patient diary data. The output data of the armband did not enable us to address the issue of whether the higher EE comes from an increase in physiological physical activity or in dyskinesias. Accurate tuning of the algorithm elaborating the accelerometer signals should be implemented to be able to distinguish between the two in subsequent studies. In any case, the improvement in PGI suggests that the increase in dyskinesias did not counteract the benefit of improved motor function. Indeed, dyskinesias do not always cause disability and actually show that levodopa is being absorbed and is effective. In addition, it is well known that fluctuating patients (motor fluctuations were an inclusion criterion) prefer dyskinesias to OFF episodes, which were another inclusion criterion, as patients had to have postprandial OFF episodes (Palmer et al. 2000).

The improvements achieved in OFF and ON time are consistent with those recorded in the previous study (Barichella et al. 2006), in which a diet with a controlled protein content (0.8 g/kg body weight) was compared with a low-protein diet based on the usage of low-protein food marketed for renal patients.

A key issue in this study is the subjectivity of the patient diary data and the novelty of the armband used for the measurement of the primary endpoint, EE. The use of patient diariesis a generally accepted method by regulatory authorities for the assessment of medicinal products (EMEA, CPMP/EWP/563/1995). Sensewear Pro2 has already been extensively used for research purposes and its use has been validated not only for usage in sports medicine and in particular environments, such as under water, but also during normal daily activity (Mignault et al. 2005; Levine and Foster 2005). Consequently, its measurement may be considered to be reliable. At present no alternative methods and devices for medium-term daily EE monitoring are available.

It would have been useful to measure the blood levels of levodopa and of the amino acids that compete with the drug, as this would enable us to understand where the competition occurs (at the blood–intestine and/or blood–brain barrier). However, the primary objective of our study was different, namely to establish whether the body weight loss that occurs during LPP consumption is due to greater EE or not; our secondary objectives were to establish whether the ON periods associated with dyskinesias increase and whether the evening meal has an influence or not. We have already planned another study focusing on amino acid/levodopa competition, in which blood levels will be measured.

Thus, the findings of this study suggest that the consumption of LPP for renal patients is a simple way to improve the therapeutic efficacy of levodopa, which does not appear to have any important drawbacks, as dyskinesias are not a major problem and malnutrition can easily be prevented by increasing calory intake. Its rationale is based on the recommendation to reduce protein intake at midday in order to prevent their interference with levodopa absorption associated with postprandial OFF episodes, which is included in international guidelines for the management of PD (Olanow 2001; Italian Neurological Society 2003). Indeed, PD patients should consume these products, which have been on the market for more thana decade, only at midday and not throughout the day as renal patients (Kopple, 2001), so the overall risk of malnutrition in PD patients is lower than in renal patients.

In conclusion, the consumption of renal LPP is associated with an improvement in motor function and an increase in EE in PD patients to a greater extent than NO-LPP dietary regimen alone. The increase in EE should be taken into account for the overall management of PD patients: calorie intake should be increased to prevent malnutrition in the long-term.
